# Predicting, Recognizing, and Treating Right Heart Failure in Patients Undergoing Durable LVAD Therapy

**DOI:** 10.3390/jcm11112984

**Published:** 2022-05-25

**Authors:** Teresa S. Wang, Marisa Cevasco, Edo Y. Birati, Jeremy A. Mazurek

**Affiliations:** 1 Division of Cardiovascular Medicine, Department of Medicine, Perelman School of Medicine, University of Pennsylvania, Philadelphia, PA 19104, USA; jeremy.mazurek@pennmedicine.upenn.edu; 2 Division of Cardiovascular Surgery, Department of Surgery, Perelman School of Medicine, University of Pennsylvania, Philadelphia, PA 19104, USA; marisa.cevasco@pennmedicine.upenn.edu; 3 Division of Cardiovascular Medicine, Padeh-Poriya Medical Center, Bar-Ilan University, Ramat Gan 5290002, Israel; ebirati@poria.health.gov.il

**Keywords:** right ventricular failure, left ventricular assist device, mechanical circulatory support

## Abstract

Despite advancing technology, right heart failure after left ventricular assist device implantation remains a significant source of morbidity and mortality. With the UNOS allocation policy change, a larger proportion of patients proceeding to LVAD are destination therapy and consist of an overall sicker population. Thus, a comprehensive understanding of right heart failure is critical for ensuring the ongoing success of durable LVADs. The purpose of this review is to describe the effect of LVAD implantation on right heart function, review the diagnostic and predictive criteria related to right heart failure, and discuss the current evidence for management and treatment of post-LVAD right heart failure.

## 1. Introduction

Durable left ventricular assist device (LVAD) technology has advanced considerably over the past 20 years, such that current third-generation devices (HeartMate 3, Abbott, Chicago, IL, USA) have an event-free survival rate of nearly 80% at two years [1,2]. However, despite improved outcomes and technology, early right heart failure (RHF) continues to be a familiar complication, with incidence estimates as high as 40% [3,4,5,6] in second-generation devices (HeartMate II and HeartWare) and 34% in current third-generation devices [2]. RHF remains a significant source of morbidity and mortality, leading to hypoperfusion, end-organ dysfunction, prolonged ICU and hospital length of stay, and increased mortality [6,7,8,9,10]. For those patients with severe RHF requiring temporary right ventricular assist device (RVAD) placement, one-year mortality is as high as 41% [6].

Historically, implantable LVADs were utilized for shorter periods of time while patients awaited availability of a donor organ (i.e., bridge-to-transplantation, BTT). Since the UNOS allocation policy changed in 2018, higher acuity patients tend to proceed directly to transplant, and stable LVAD patients have significantly longer wait times [11,12]. As a result, the phenotype of patients receiving LVADs seems to have shifted, with a larger proportion of destination therapy (DT) implants and an overall sicker population. In this sicker contemporary population, RHF will continue to pose a significant obstacle despite technological innovation, and a comprehensive understanding of the timing, predictors, and treatment of RHF will be critically important in the ongoing success of LVADs.

We aimed to describe the interplay of right ventricular function with LVADs; review the diagnostic criteria as well as clinical, imaging, and hemodynamic factors related to RHF; discuss the current evidence for management and treatment strategies to handle this challenging entity.

## 2. Right Heart Function in LVADs

### 2.1. Anatomy and Function of the Right Heart

The right ventricle (RV) is distinct from its left ventricular (LV) counterpart as a thin-walled and heavily trabeculated triangular structure. Within the RV sits the crista supraventricularis, a muscle bridge dividing the tricuspid valve inflow from the pulmonic valve outflow. The crista supraventricularis plays a crucial anatomic role in RV function, as its muscle fibers interlace with the interventricular septum (and thus, the LV) and the RV free wall; during systole it acts to narrow the tricuspid orifice and contract the RV free wall towards the interventricular septum [13,14].

The purpose of the RV is threefold: (1) to preserve low systemic venous pressures to avoid liver and kidney dysfunction, (2) to maintain forward momentum of blood flow to the pulmonary circulation, and (3) to provide oxygenated preload to the left heart in order to maintain adequate perfusion [14,15,16]. In contrast to the LV, the RV pumps to a low-impedance and highly compliant pulmonary circulation, which is reflected in the absence of isovolumic phases of contraction and relaxation in RV pressure-volume loops [14,16,17].

The RV is influenced by multiple factors including systemic venous return (preload), pulmonary resistance (afterload), and intrinsic contractility. Under optimal conditions, the RV cardiac output approximates that of the LV; this is also affected by ventricular interdependence, with the LV contributing to RV output via the septum. Importantly, RV function is sensitive to afterload, which can be defined as the RV wall stress during systole as estimated by a combination of attributes (resistance, compliance, arterial wave reflections, inertance of blood) during systolic ejection [14,18]. As afterload increases, RV stroke volume declines resulting in greater energy expenditure. This phenomenon of decreased mechanical efficiency and increased oxygen consumption to maintain adequate RV output is termed right ventricular-pulmonary artery (RV-PA) uncoupling and can be a harbinger for adverse outcomes [19,20].

### 2.2. Influence of VAD on Right Heart Function

**Figure 1:**
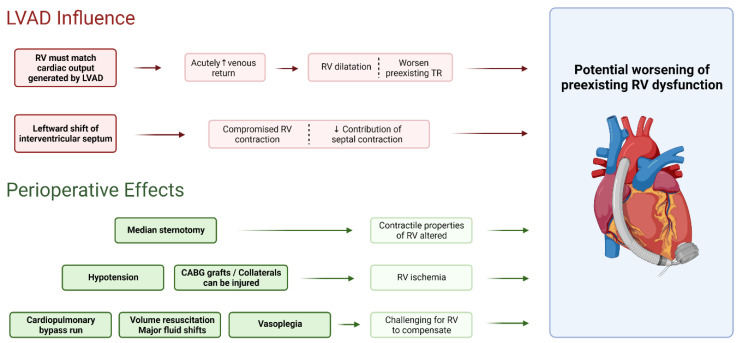
Influence of left ventricular assist device and perioperative factors on right heart function. CABG = coronary artery bypass graft; LVAD = left ventricular assist device; RV = right ventricle; TR = tricuspid regurgitation; ↑ = increased; ↓ = decreased. Created with BioRender.com, accessed on 22 April 2022.

LVAD implantation results in an immediate and dramatic shift in hemodynamics and physiology. As the LVAD decompresses the LV, there is an improvement in upstream pressures (reduction in left atrial and pulmonary artery pressures) and mitral regurgitation. This results in decreased RV afterload due to the improved LV filling pressures and the theoretical expectation of improved RV function. However, there are multiple forces at play that may, in fact, lead to RV dysfunction and subsequent failure (Figure 1). While cardiac output normalizes with LVAD implantation, there is acutely increased venous return to the right heart compared to pre-LVAD implantation, and the RV must match the cardiac output generated by the LVAD which may exacerbate pre-existing RV dysfunction [15,21,22]. RV dilatation due to the increased preload may worsen preexisting tricuspid regurgitation. LVAD flow may also lead to leftward shift of the interventricular septum with subsequent compromise of RV contraction due to the changes in RV shape (more spherical) and reduced contribution of septal contraction. Moreover, contractile properties of the RV change following median sternotomy, with a shift from longitudinal to transverse shortening [23,24,25].

Intraoperative factors also have a significant impact on RV function and particularly early RHF [24]. Cardiopulmonary bypass runs challenge the RV, hypotension may result in RV ischemia, and coronary artery bypass grafts or collaterals can be injured. Blood products given during the implant can further increase the risk of early RHF [26], and volume resuscitation can result in major fluid shifts and worsen RV dilatation, wall stress, and tricuspid regurgitation. Vasoplegia in the postoperative period may require the use of inotropes or vasopressors, thereby increasing myocardial demand and potentially exceeding the RV’s ability to compensate.

Studies analyzing the effects of LVAD implantation on RV hemodynamics have demonstrated that pulmonary vascular resistance (PVR) decreases dramatically in the first three months post-VAD implantation [25,27]. This is an expected hemodynamic effect early after acutely unloading the left side of the heart. Beyond three months, however, there is a smaller and more modest decrease in PVR long term. [27,28]. Unfortunately, RV adaptation to load worsens after LVAD implantation, and this remains constant over time, suggesting increased afterload sensitivity of the RV following LVAD implantation [25]. However, RV load can improve with more prolonged support and, thus, RV performance may still improve over time.

## 3. Diagnosis and Recognition of Right Heart Failure

### 3.1. Definitions of Early and Late Right Heart Failure

Definitions for post-LVAD right heart failure vary significantly in clinical criteria and time frame but generally include the need for intravenous inotropes, pulmonary vasodilators, and/or right-sided circulatory support [3,4,6,29,30,31,32,33,34] (Table 1). Some early studies investigating RHF in pulsatile-flow and axial-flow devices tended towards more narrow criteria requiring the need for RVAD implantation [30,32,33]; others were more inclusive, defining RHF as the need for postoperative inotropic support for >14 days, pulmonary vasodilators for ≥48 h, and/or need for right-sided circulatory support [29,31]. Kormos et al. described RHF as early and late, and differentiated the two due to the ostensibly different pathophysiologic mechanisms; early RHF was defined by the need for RVAD and/or continuous inotropic support for ≥14 days post-implantation, while late RHF was defined as the need for inotropic support starting 14 days after implantation [6].

In 2014, the Interagency Registry for Mechanically Assisted Circulatory Support (INTERMACS) proposed a more granular definition of post-LVAD RHF using hemodynamics and clinical findings [35]. RHF was defined as elevated central venous pressure (right atrial pressure > 16 mmHg, dilated inferior vena cava on echocardiography, or elevated jugular venous pressure) along with manifestations of elevated central venous pressure (i.e., peripheral edema, ascites or hepatomegaly, and laboratory evidence of worsening hepatic or renal dysfunction). It was further divided by severity based on duration of inotropes, inhaled nitric oxide, or intravenous vasodilators: mild—weaned within 7 days; moderate—weaned within 7–14 days; severe—surpassing 14 days or need for RVAD. However, this definition has not become universally accepted as subsequent risk scores, and clinical trials continue to utilize variations of this definition. In 2017, the EUROMACS Right Heart Failure Risk Score defined RHF as receiving short- or long-term right-sided circulatory support, continuous inotropic support for ≥14 days, or nitric oxide ventilation for ≥48 h [3]. Different still, the MOMENTUM 3 clinical trial of HeartMate 3 LVAD defined RHF as the need for RVAD implantation, inhaled nitric oxide, or inotropic agents for >7 days at any time following LVAD implantation [34].

Late RHF is even less well defined, as many definitions combine early and late RHF into one category. However, late RHF is generally determined to occur after initial hospital discharge [14,24] (Table 1). Kormos et al. and Loghmanpour et al. defined the time frame as occurring 14 days after LVAD implantation, but Takeda et al. differed by extending the time frame and defining late RHF as the need for rehospitalization following index hospitalization [4,6,10]. Rich et al. defined it as occurring >30 days after discharge from the index hospitalization [8].

The lack of standardized definitions for early and late RHF is problematic as reproducibility is limited, valid comparisons of incidence rates are challenging, and prediction models demonstrate significant variability [36]. Utilization of a universally accepted definition will allow a more accurate understanding of incidence rates and risk factors, validation of prediction risk scores, and ability to better phenotype this patient population.

### 3.2. Risk Models

Numerous risk scores and predictive models have been created to better identify patients at risk for post-LVAD RHF. The goals of these models are to gain insight into patient prognosis, improve risk stratification and patient selection, and allow for implementation of treatment strategies to avoid poor outcomes [15,22,36]. Models able to distinguish low- versus high-risk patients will therefore be especially valuable and affect patient outcomes. However, all models have inherent limitations making accurate identification of this patient population challenging.

Many existing risk score models were derived from single-center cohorts [29,30,31,32,33], some without validation in external cohorts therefore limiting the generalizability of these models. Furthermore, risk scores were studied in patients implanted with pulsatile or early-generation continuous flow devices, thus limiting applicability. Critical appraisal of these risk models show that discrimination in validation cohorts is modest at best with a C-statistic reaching only 0.65 [36]. As discussed above, the lack of a universally accepted definition for RHF led to variability in the prediction models; particularly problematic is the arbitrary use of time duration. Moreover, wide variation in institutional thresholds for early versus delayed RVAD use may introduce significant unwanted bias [22]. As a result, uptake and application of these risk scores in clinical practice has been limited. However, a comprehensive understanding of the factors incorporated into risk models is nonetheless necessary.

### 3.3. Risk Factors

**Figure 2:**
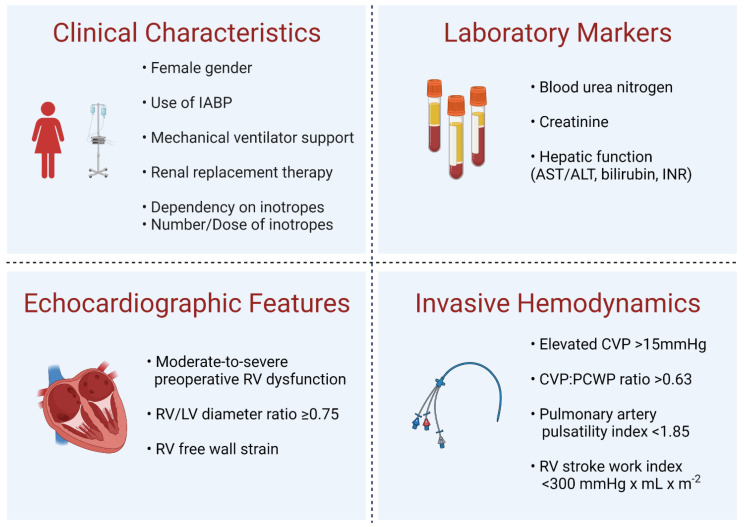
Risk factors associated with post-LVAD right heart failure: CVP = central venous pressure; IABP = intra-aortic balloon pump; LV = left ventricle; PCWP = pulmonary capillary wedge pressure; RV = right ventricle. Created with BioRender.com, accessed on 22 April 2022.

There are a host of clinical characteristics, laboratory values, imaging data, and hemodynamic parameters associated with post-LVAD RHF (Figure 2). In a large meta-analysis, female gender, use of intra-aortic balloon pump, mechanical ventilatory support, and use of renal replacement therapy were all found to be independent predictors of early RHF with odds ratios (OR) ranging 1.59–4.61 [21]. Inotrope or vasopressor requirement, particularly dependency and number of inotropic agents, are also utilized in multiple risk prediction scores [3,29,31,32]; it has been shown that longer duration of milrinone infusion has been associated with higher prevalence of RHF with an odds ratio of 6.3 [37]. Laboratory markers of end organ damage are also frequently implicated as risk markers for RHF, especially renal (i.e., serum blood urea nitrogen and creatinine) and hepatic function (i.e., transaminases, bilirubin and international normalized ratio) [21,29,30,32]. However, it is important to note that poor preoperative renal and hepatic function is not necessarily specific to RHF and may also reflect overall end-stage or critical illness [36]. NT-proBNP was observed to be higher in patients with RHF, though it was highly heterogenous [21,38].

Echocardiography is an enticing modality to determine both accurate preoperative RV function and factors that may predict post-LVAD RHF. However, technical challenges (i.e., LVAD-related artifact), poor reproducibility, and low interobserver correlation make reliance on echocardiography more challenging. Interestingly, only the Penn RVAD, CRITT, and EUROMACS-RHF risk scores utilize preoperative RV dysfunction on echocardiography in their models [3,30,33]. Meta-analysis did show that moderate to severe RV dysfunction (assessed qualitatively) was associated with higher risk of RHF (OR 2.82) [21], though performance of these specific models was still modest at best [36]. Tricuspid annular plane systolic excursion (TAPSE) has not borne out as a predictive factor due to the fact of its heterogeneity and dependence on loading conditions [39,40,41]; it also does not account for the septal contribution to RV function. Tissue doppler imaging for the systolic velocity (S’) of the tricuspid annulus could pose an alternative to TAPSE, with a small study showing lower preoperative S’ in LVAD patients who developed RHF [42]. However, this measure is still dependent on insonation angle and loading conditions, and may not be representative if regional abnormalities are present [22]. The more quantitative measure of RV/LV diameter ratio, a surrogate for RV function, has been demonstrated in multiple studies to be predictive of RHF [21,43,44]. Specifically, an RV/LV ratio ≥ 0.75 was independently associated with RHF and 30 day mortality and was additive for existing risk scores [43,44]. RV fractional area change (RVFAC) is another quantitative measurement that could be useful, with a cutoff of <35% considered abnormal. However, utility of this measure in predicting RHF is unclear, and reproducibility and availability of the software package are known challenges [22,40]. RV free wall strain has been predictive of RHF following LVAD implantation [38,41,45,46] and is a promising marker for clinical use.

Invasive hemodynamics are crucial in the assessment of RV function and proper risk stratification prior to LVAD implantation. A large study of 475 LVAD patients showed that elevated central venous pressure (CVP) was an independent predictor of postoperative RHF [47], with the largest effect size in patients with continuous flow devices [21]. Elevated CVP > 15 mmHg and increased ratio of CVP to pulmonary capillary wedge pressure (PCWP) > 0.63 have been identified as risk factors in multiple predictive models [3,6,32,33]. Kormos et al. showed that a CVP/PCWP ratio of >0.63 was associated with an OR of 2.5 and helped differentiate those needing RVAD support from those requiring prolonged inotropic support. The calculated pulmonary artery pulsatility index (PAPi) has also been shown as a robust predictor of postoperative RHF, as it combines the pulmonary artery pulsatility in the numerator and the right atrial pressure in the denominator to gain insights on RV loading conditions [38,48,49]. Morine et al. found a PAPi < 1.85 resulted in an area-under-the-curve of 0.942, surpassing the test characteristics of other hemodynamic markers mentioned above [48]. Kang et al. demonstrated that higher PAPi, and therefore more robust RV function, was associated with lower rates of RVAD implantation (OR 0.31) [49]. Pulmonary arterial compliance combined with CVP:PCWP ratio has also been shown to identify patients at high risk for RHF and 6 month mortality [50]. Another calculated variable, RV stroke work index (RVSWI), is a load-dependent contractile index that is a significant determinant of postoperative RHF, particularly when RVSWI < 300 mmHg × mL × m^−2^ [6]. Notably, RVSWI was a powerful discriminator in pulsatile devices [21], though this has not been consistent for continuous flow devices [48].

Risk factors for late RHF differ slightly from those mentioned above. Two large single-center studies examined predictors for late RHF and found that typical clinical characteristics for early RHF, such as intra-aortic balloon pump use, mechanical ventilatory use, or use of vasopressors or inotropic support, were, in fact, not predictive of late RHF. Pump speed and even the occurrence of early RHF were not predictors. Rich et al. found that elevated preoperative BUN and increased CVP:PCWP ratio were independent predictors of late RHF, while Takeda et al. found that diabetes, BMI > 29, and elevated BUN were univariate predictors for late RHF [8,10].

## 4. Management of Right Heart Failure

### 4.1. Preoperative Medical Management

Management of RHF preoperatively should utilize a multifaceted approach aimed at addressing RV preload, afterload, and contractility. RV preload should be optimized aggressively, understanding that the patient will likely undergo multiple blood product transfusions intraoperatively. While there is no standard CVP target used, ideally the CVP goal should be <15 mmHg given that a CVP > 15 mmHg is associated with post-LVAD early RHF [21,49]. Hemodynamic monitoring with a central venous catheter is encouraged if the patient’s volume status is uncertain. Ultrafiltration can be considered if refractory to high-dose diuretics [14].

Mitigating causes of elevated PVR is also an important goal given that the RV is particularly sensitive to afterload. Use of inhaled pulmonary vasodilators like epoprostenol and nitric oxide are advantageous in selected patients as it avoids systemic hypotension and has beneficial short-term effects in lowering PVR and improving RV cardiac output [51,52]. Sildenafil, a phosphodiesterase-5 inhibitor (PDE5i), is used not infrequently as an off-label medication to alleviate secondary pulmonary hypertension in end-stage heart failure patients awaiting LVAD implantation [53]. However, a retrospective multicenter study of LVAD recipients found that preoperative PDE5i use was associated with adverse outcomes including increased incidence of severe early RHF and increased risk of major postoperative bleeding [54].

It is also crucial to enhance forward flow and augment RV perfusion. If inotropes are used, milrinone and dobutamine are the preferred agents given their combined inotropic and vasodilator properties. Milrinone results in both pulmonary and systemic vasodilation with increased myocardial inotropy, ultimately serving to reduce both RV and LV end-diastolic pressures; thus, milrinone is the preferred agent over dobutamine in appropriate situations [14]. However, it must be noted that milrinone may trigger hypotension, has a longer half-life, and is partially renally cleared.

### 4.2. Intraoperative Management

There are a number of perioperative factors that can impact RV function postoperatively including cardiopulmonary bypass time, perioperative bleeding, volume loading with blood product transfusions, interventricular septal shift once the LVAD is implanted, and disruption or injury of blood flow to the right coronary artery (bypass grafts or collaterals) [15,24,55]. Minimizing bypass time, bleeding, blood product transfusions, and optimizing pump speed to avoid septal shift are all critical interventions that may mitigate the incidence of early RHF.

Median sternotomy has been the standard surgical approach for LVAD implantation, with obvious advantages including full visualization and ability to perform other interventions and place central RV support if necessary. An alternative lateral thoracotomy approach was first studied in the HeartWare LVAD, and showed that it was a safe and effective alternative in selected patients [56]. Advantages included maintaining chest stability, decreased bleeding and need for blood product transfusions, and earlier recovery. Currently, the SWIFT trial is actively enrolling patients to investigate implantation of the HeartMate 3 LVAD by thoracotomy, and it is expected to complete enrollment by mid-2022 [57].

There is lack of consensus regarding the utility and efficacy of concurrent tricuspid valve repair (TVr) for functional tricuspid regurgitation. Early observational studies suggested that concomitant TVr improved early clinical outcomes, reduced incidence of early RHF, and was a durable adjuvant procedure [58,59,60]. However, more contemporary studies of larger cohorts showed that concomitant TVr did not confer any survival benefit and in fact, failed with a recurrence rate of 38% at two years follow up [61,62,63,64,65]. Moreover, Barac et al. found that concomitant TVr was an independent risk factor for the development of late RHF in their larger single-center study [63]. Thus, it appears that concomitant TVr has limited durability and centers should be cautioned against routine use of this procedure.

### 4.3. Postoperative Management

Recognition of RHF in the immediate postoperative period can be tricky, as the clinical presentation mimics that of cardiac tamponade. Oftentimes, patients return from the operating room on multiple vasopressors for vasoplegia-mediated hypotension, and echocardiography may not be informative given technical limitations in a postoperative patient. Ultimately, there are five main aspects to consider when managing the RV postoperatively: (1) preload optimization, (2) inotropy, (3) chronotropy, (4) pulmonary vasodilation and management of the PVR, and (5) optimization of the systemic mean arterial pressure and pump speed to support the septum. Goals are to support the patient to preserve euvolemia with an approximate CVP < 12 mmHg, maintain perfusion with a mean arterial pressure > 70 mmHg and cardiac index > 2.2 L/min/m^2^, and ensure adequate LV unloading via the device [15,24]. Adjustment of pacemakers when available may also help support the RV. Early recognition of bleeding and the need to return to the operating room for a washout are crucial as well. Close monitoring of end organ function, urine output, and lactate production will allow for timely intervention and escalation of therapies as necessary. Notably, however, data for treatment and management of RHF is limited, and there are no randomized trials that show benefit for the various perioperative and postoperative therapies.

In contrast to outcomes associated with preoperative PDE5i use, postoperative PDE5i use may in fact be beneficial with lower all-cause mortality and fewer ischemic strokes in an observational study of patients implanted with centrifugal flow LVADs; however, the mechanism by which this occurs is unclear [66]. Furthermore, identification of decoupling between diastolic pulmonary artery pressure and pulmonary capillary wedge pressure (i.e., an index of pulmonary vascular damage) may be useful in prediction of outcomes. Imamura et al. found that decoupling of the diastolic pulmonary artery pressure gradient is a strong predictor of death or heart failure readmission, in addition to worsening of right heart function [67,68].

### 4.4. Mechanical Support

Early consideration and institution of mechanical RV support confers survival benefit and helps to prevent potentially irreversible multiorgan failure [9,69,70]. In a large single center study, unplanned RVAD support resulted in high rates of mortality, with even poorer outcomes in those patients unable to be weaned from RVAD support [9]. Thus, appropriate *preoperative* patient selection may help to identify patients who would benefit from planned or early institution of biventricular support.

Options for mechanical RV support include both temporary and intermediate devices [14,71,72]. Temporary device options include: (1) Impella RP (Abiomed Inc., Danvers, MA, USA), (2) PROTEK Duo (LivaNova, London, UK), and (3) veno-arterial extracorporeal membrane oxygenation (VA-ECMO). The Impella RP is a recently FDA-approved (for right heart support) microaxial device that is placed percutaneously via the femoral vein to pump blood from the right atrium (RA) through to the PA [73]. However, challenges exist with this device including postoperative bleeding and malposition. The PROTEK Duo is a dual-lumen cannula placed percutaneously via the internal jugular vein to drain blood from the RA and shuttle it to the PA [74]. Advantages include ease of insertion and ability for patients to ambulate.

Intermediate options for mechanical RV support involve surgical implantation of an RVAD with central cannulation of the RA or RV for venous inflow and the PA for arterial outflow. The cannulas are connected to centrifugal flow pumps such as CentriMag Pump (Abbott), RotaFlow (Macquet CP), and TandemHeart (LivaNova). Unfortunately, long-term options for durable RVADs do not exist and remain a thorn that limits the success of durable LVADs.

## 5. Future Directions and Conclusions

Despite an improvement in our understanding of RHF over the years, this common complication continues to present a considerable challenge in the overall success of durable LVADs. A basic and necessary step moving forward will be to agree on a standardized definition of RHF. In our opinion, early RHF may be best defined by the MOMENTUM criteria, as this will be most relevant for third-generation devices. For late RHF, we agree with the definition, set forth by Takeda et al., as rehospitalization following index admission [10]. Much like the universal definition of heart failure [75], a universally accepted definition for RHF would be specific yet clinically relevant and allow for improvements in clinical care, registry research, and clinical trials.

As the landscape of referrals to durable LVAD change following the 2018 UNOS allocation policy change, we must also enhance our approach to patient selection. Registry data show that patients with INTERMACS profile 3+ tend to fare better than those classified as INTERMACS 1 or 2 [76,77]. Strategies to optimize patients preoperatively and determine appropriate timing to go to LVAD will be the holy grail in our ability to improve patients’ quality of life and survival.

**Figure 3:**
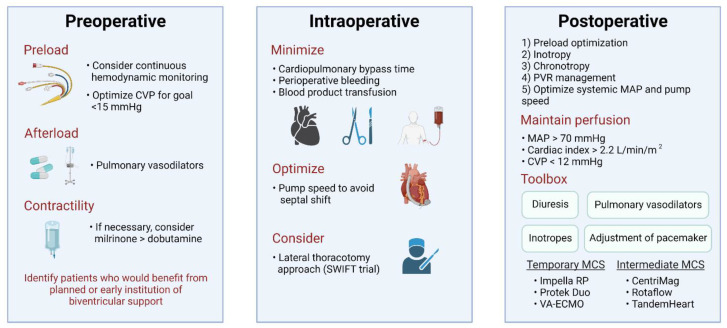
Management of right heart failure with left ventricular assist device placement. CVP = central venous pressure; LV = left ventricle; MAP = mean arterial pressure; MCS = mechanical circulatory support; PVR = pulmonary vascular resistance; VA-ECMO = veno-arterial extracorporeal membrane oxygenation. Created with BioRender.com, accessed on 22 April 2022.

We will need to continue to push the boundaries of therapies in the management of RHF following LVAD implantation. While we highlight a systematic strategy to approach the postoperative LVAD patient (Figure 3), we will need to be creative in affecting existing pathophysiological processes for new therapeutics. For example, Uriel et al. tested the use of CRD-102 (novel oral extended release formulation of milrinone) in the treatment of chronic RHF in LVAD patients with successful proof-of-concept results [78]. Innovations on existing surgical techniques and perioperative management will be intriguing, and we eagerly await the results of the SWIFT trial in the future. Lastly, despite our rapid technological advances over the past 20 years, there remains a large unmet need for biventricular device support, particularly durable right-sided support. Further studies are needed to succeed in the recognition, prediction, and treatment of RHF in the LVAD population.

## Figures and Tables

**Table 1:** Varying definitions of early and late right heart failure following LVAD implantation.

**Author**	**Year**	**Definition**	**Severity Distinction**
Early Right Heart Failure			
Matthews et al. [29]	2008	Need for IV inotropes >14 days, inhaled nitric oxide ≥48 h, right-sided circulatory support (ECMO or RVAD) or hospital discharge with IV inotrope	
Fitzpatrick et al. [30]	2008	Need for RVAD support	
Kormos et al. [6]	2010	Need for RVAD or continuous inotropic support ≥14 days following implantation	
Drakos et al. [31]	2010	Need for RVAD implantation, inhaled nitric oxide ≥48 h, or need for IV inotropes >14 consecutive days	
Wang et al. [32]	2012	Need for RVAD support	
Atluri et al. [33]	2013	Need for RVAD support	
INTERMACS [35]	2014	Elevated CVP (RAP > 16 mmHg, dilated IVC on echocardiogram, or elevated jugular venous pulse) + manifestations of elevated CVP (peripheral edema, ascites/hepatomegaly, lab evidence of worsening hepatic or renal dysfunction)	Mild: within 7 days Moderate: 7–14 days Severe: >14 days or need for RVAD
Soliman et al. [3]	2018	Short- or long-term right-sided circulatory support, continuous inotropic support ≥14 days, or inhaled nitric oxide ventilation ≥48 h	Severe
Rich et al. [8]	2017	RV dysfunction associated with signs/symptoms of RHF * that warrant RVAD, use of inotropes >14 consecutive days, or need to reinitiate inotropes between 14 and 30 days post-implant	
Mehra et al. (MOMENTUM 3) [34]	2019	Signs/symptoms of persistent RV dysfunction * requiring RVAD, inhaled nitric oxide, or inotropes >7 days anytime following LVAD implantation	
Late Right Heart Failure			
Kormos et al. [6]	2010	Inotropic support starting >14 days post-implantation	
Takeda et al. [10]	2015	Right heart failure requiring rehospitalization following index hospitalization in addition to medical/surgical management (i.e., augmented diuretics, inotropes, and RVAD implantation)	
Rich et al. [8]	2017	RV dysfunction associated with signs/symptoms of RHF * that warrant readmission with initiation of inotropes >30 days following discharge from index hospitalization	

* Signs/symptoms of RV dysfunction or right heart failure include peripheral edema, ascites/hepatomegaly, and lab evidence of worsening hepatic or renal dysfunction. CVP = central venous pressure; ECMO = extracorporeal membrane oxygenation; IV = intravenous; RAP = right atrial pressure; RVAD = right ventricular assist device.
